# Chromosomal rearrangements: tempo and mode of karyotype evolution in Scarabaeoidea

**DOI:** 10.1093/jeb/voag025

**Published:** 2026-04-09

**Authors:** Sean Chien, Heath Blackmon

**Affiliations:** Department of Biology, Texas A&M University, College Station, United States; Department of Biology, Texas A&M University, College Station, United States; Ecology and Evolutionary Biology IDP, Texas A&M University, College Station, United States

**Keywords:** chromosome number, fusion, fission, sex chromosome–autosome fusion, karyotype, Coleoptera

## Abstract

Karyotype evolution, including changes in chromosome number and structure, is a fundamental process that can influence genome organization and speciation. Here, we analyze patterns of chromosome number evolution and sex chromosome–autosome (SA) fusions across Scarabaeoidea, a diverse beetle superfamily with broad variation in karyotypes and sex chromosome systems. We compiled 478 karyotype records and constructed a phylogeny for 211 species to estimate rates of chromosome number change, modeled as fission and fusion events, in Scarabaeidae, Lucanidae, and Passalidae. Passalidae show markedly higher rates of both fission and fusion along with consistently higher chromosome numbers. This pattern is consistent with genetic drift facilitating the fixation of rearrangements in lineages with low dispersal and small effective population sizes. We also tested whether SA-fusions occur more often than expected by chance, where the null expectation assumes all chromosomes are equally likely to undergo fusion. The observed proportion of SA-fusions was more than double the null expectation, with non-overlapping credible intervals. This pattern is consistent with a prediction of models where sex chromosome fusions are indirectly favored by sexually antagonistic selection. We also found that neo-XY systems are consistently associated with lower autosome numbers, consistent with repeated SA-fusions. Together, these results show that Scarabaeoidea exhibit dynamic karyotype evolution and provide comparative support for core predictions of the canonical model of sex chromosome evolution.

## Introduction

Karyotype evolution, the process by which chromosome number and structure change over time, represents one of the most fundamental aspects of genome evolution. Even before the chromosomal theory of inheritance was developed, the study of karyotypes offered a first glimpse into the organization of genetic material (Flemming, 1882; Sutton, 1903). Despite rapid advances in DNA sequencing, karyotype data remain a critical window into the forces shaping genome evolution (Hjelmen, 2024), allowing us to understand patterns of chromosomal diversification across lineages (Anderson et al., 2020; Blackmon et al., 2024; Deakin et al., 2019; Mayrose & Lysak, 2021). While classical karyotyping reveals macro-scale patterns of evolution, the integration of molecular cytogenetics and cytogenomics, including comparative mapping and chromosome painting, is essential for identifying conserved synteny and structural evolution. These disciplines bridge the gap between physical chromosomes and chromosome-level genome assemblies, providing a framework to validate genomic data and explore the functional genomic consequences of rearrangements (Deakin et al., 2019; O’Connor et al., 2017; Saunders et al., 2025). Yet, a key gap remains in our understanding of how frequently chromosomal rearrangements shape genome evolution across diverse clades.

Changes in karyotype are driven by both chromosomal rearrangements, including fissions, fusions, translocations and changes in chromosome number, such as whole-genome duplication, and aneuploidy. For example, fusions, such as Robertsonian translocations, can merge two acrocentric chromosomes (Schubert & Lysak, 2011), while centric fissions split a chromosome in two. This process typically occurs via a break in the centromeric region, but can also arise from a non-centromeric break if the resulting acentric fragment is stabilized, for example by acquiring new telomeres and a neocentromere (Harrington & Greider, 1991; Tsujimoto et al., 1999). Empirical evidence from diverse eukaryotes indicates that epigenetic remodeling can establish new centromeric activity on acentric fragments rapidly enough to ensure mitotic stability and retention in the karyotype (Burrack & Berman, 2012; Scott & Sullivan, 2014). Whole-genome duplication events, as well as deviations in chromosome number due to aneuploidy, can further remodel the genome (Orr et al., 2015). These large-scale genome reorganization events have broad implications because by altering recombination patterns and the strength of genetic linkage, they can modulate the genomic landscape of diversity and selection, ultimately impacting reproductive isolation and speciation (Guerrero & Kirkpatrick, 2014; Huang & Rieseberg, 2020; Lucek et al., 2023; Muller, 1916; Ostberg et al., 2013; Ross et al., 2015; Sturtevant, 1917; Vara et al., 2021; Yoshida et al., 2023).

Increases in chromosome number through fission, for example, may elevate recombination rates, whereas chromosomal fusions generally reduce them. This reduction occurs not only by suppressing crossing-over in structural heterozygotes but also altering the recombination landscape in homozygotes through crossover interference and physical changes in chromosome architecture (Dumas et al., 2015; Guerrero & Kirkpatrick, 2014; Vara et al., 2021; Yoshida et al., 2023). These processes interact with other evolutionary forces, including effective population size and meiotic drive, creating lineage-specific rates of karyotype evolution (Blackmon et al., 2019, 2024; Jonika et al., 2024; Robinson, 1995; Searle & Pardo-Manuel de Villena, 2024; Sylvester et al., 2020). In smaller populations, for example, genetic drift can facilitate the fixation of mildly deleterious chromosomal changes (Lande, 1985; Wright, 1941), whereas lineages with highly conserved karyotypes may be constrained by strong purifying selection against new rearrangements (Rieseberg, 2001). These rearrangements often cause pairing and segregation problems during meiosis in heterozygotes, leading to reduced fertility (Blackmon et al., 2019).

Sex chromosomes have long fascinated evolutionary biologists because they experience fundamentally different evolutionary forces than autosomes with predictable differences in population size, exposure to selection in male vs female backgrounds, and ploidy (Larson et al., 2018; Sackton et al., 2014). Classic theory predicts that fusions between autosomes and sex chromosomes could be selectively favored for their ability to resolve sexual antagonism and ensure proper chromosome pairing and segregation during meiosis (D. Charlesworth & Charlesworth, 1980). Yet only recently have probabilistic models enabled rigorous tests of whether these fusions are more common than expected by chance (Anderson et al., 2020; Yoshida & Kitano, 2021) (Anderson et al., 2020).

In this context, the superfamily Scarabaeoidea provides a compelling system for studying karyotype and sex chromosome evolution. Scarabaeoidea is exceptionally diverse, well-studied, and exhibits a wide range of sex chromosome systems and autosome numbers (A.-M. Dutrillaux & Dutrillaux, 2012). Here, we synthesize karyotype data and phylogenetic analyses for Scarabaeoidea to quantify patterns and rates of chromosome number change and to evaluate whether sex chromosome–autosome fusions are overrepresented relative to a null model where fusions occur randomly among all available autosomes and sex chromosomes. Our results reveal striking variations in rates of chromosome evolution across families within Scarabaeoidea and demonstrate that fusions involving sex chromosomes occur more frequently than expected. This provides empirical evidence from a highly diverse clade with exceptional karyotypic variation supporting models of sex chromosome evolution that rely on sexually antagonistic selection.

## Methods

### Data collection

We downloaded karyotype data from the Coleoptera Karyotype Database (Blackmon & Demuth, 2015). This dataset collects karyotype information from numerous original studies, the vast majority of which used classical cytogenetic techniques. Therefore, the available data is primarily limited to chromosome counts and sex chromosome system identification, without information on chromosome homology or synteny across species. Sequencing data were downloaded using PhylotaR, an R package that facilitates the downloading and clustering of sequencing data from NCBI based on a provided taxonomic ID (Bennett et al., 2018). Preliminary results identified 4 clusters with the largest number of sequences available for Scarabaeoidae (taxonomic ID: 7041). These clusters were selected, and this pipeline was used to download sequencing data. Due to limited karyotype data availability, sequencing data for Lucanidae, Passalidae, Trigidae, and Glaphyridae were manually obtained from NCBI. Finally, in some cases, a genus may contain one species with karyotype data and another species with sequencing data. To maximize the power of our analyses, these were both retained as genus-level matches. The final supermatrix dataset includes two fragments of cytochrome oxidase subunit I (COI), 16s rRNA, and 28s rRNA. Genus-level tips were used for 37 taxa in our final dataset (36 in Scarabaeidae, 1 in Lucanidae, and 1 in Passalidae). While this method increases taxon sampling, it can also introduce phylogenetic uncertainty. To assess the potential impact of this data imputation on our conclusions, we conducted another analysis by repeating our main analyses on a reduced dataset that excluded these 37 genus-level representatives.

### Phylogenetic inference

MAFFT version 7 was used to align our four sequence sets separately (Katoh et al., 2019). For the RNA sequencing datasets, we used E-INS-i as the iterative refinement method with all default settings, and for the mitochondrial DNA sequencing datasets, we used G-INS-1 as the iterative refinement method with 0.8 unaligned level. After alignment, Seqotron was used to manually check alignment quality and remove low-quality alignments (Fourment & Holmes, 2016). Additionally, Gblocks was used with default parameters to remove hypervariable regions in the 16s and 28s alignments (Castresana, 2000). Finally, all datasets were combined into a supermatrix using the function SuperMatrix function in the R package evobiR prior to phylogenetic inference (Jonika et al., 2023).

First, RAxML 8.2.12 was used with rapid bootstrapping to produce 500 trees under the GTR model (Stamatakis, 2014). These 500 trees were utilized to calculate the Taxonomic Instability Index (TII) for each taxon using Mesquite v. 3.81(Maddison & Maddison, 2007). A higher TII indicates higher uncertainty of a taxon placement across trees. We inspected the TII distribution visually and found that 97% of taxa have indices less than 20,000. Above 20,000, the indices increased rapidly, so we removed those 6 taxa with indices above 20,000 (Figure S1). After the removal of high TII taxa from our alignment, BEAST v. 2.6.7 was used to infer phylogenies (Bouckaert et al., 2019). The best-scoring ML tree from RAxML served as the starting tree. We inferred phylogenies under the GTR model of DNA evolution, a birth-death model for the branching structure of the tree, and a random local clock. A normal prior was applied to the node representing the MRCA of Scarabaeoidae. This normal prior had a mean of 1.837 and a standard deviation of 0.143, putting the units of branch length into hundreds of millions of years (McKenna et al., 2019). All Scarabaeoidae were set to monophyletic, forcing *Enochrus falcarius* to be the outgroup (Bouckaert et al., 2019). Parameters sampled by the MCMC run were recorded every 10^4^ generations, and Tracer v. 1.7.2 was used to check the effective sample size (ESS) of model parameters (Rambaut et al., 2018). MCMC run was terminated once ESS values exceeded 200. The final 50% of MCMC runs were retained as the posterior distribution. We sampled 100 trees from the posterior distribution to use for downstream analysis. Taxa belonging to Glaresidae, Glaphyridae, and Trogidae were removed from analyses because each was represented by less than three species in our dataset. One potential problem in comparative analyses is that misplaced taxa can lead to inflation of estimated transition rates. To guard against this we next removed any taxa whose placement on the tree conflicted with widely accepted taxonomy. Specifically, taxa were pruned from all phylogenies if their phylogenetic placement was discordant with their assigned genus-level classification on any sampled phylogeny. These discordant taxa were removed rather than recategorized. Recategorization was not appropriate for two key reasons. First, such phylogenetic discordance often signals sample misidentification, and reassigning the taxon would risk incorrectly pairing genetic data from one species with karyotype data from another. Second, retaining a misplaced taxon necessarily creates invalid branch lengths in the phylogeny, which can severely bias branch-length dependent estimates of evolutionary rates.

### Chromosome number evolution

**Figure 1:**
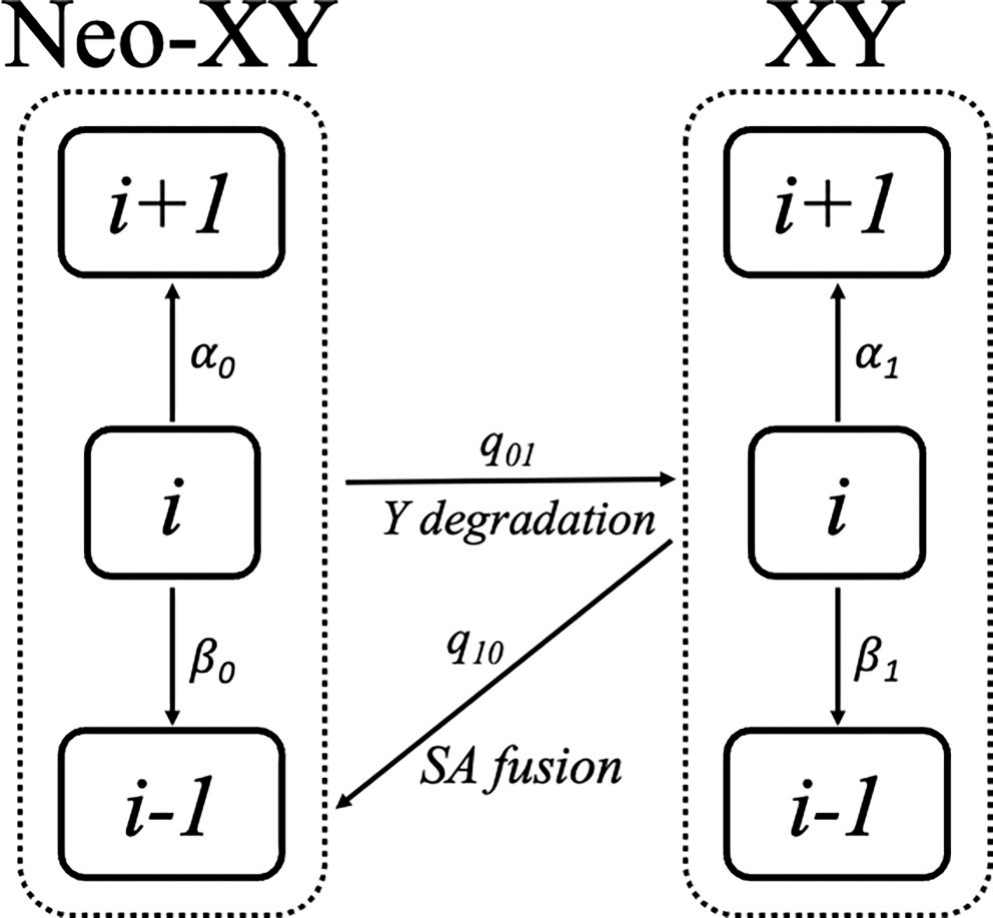
Markov model of chromosome number and sex chromosome system evolution. Chromosome numbers can change via fusion or fission within the state. Fission will increase chromosome number by one; fusion will decrease chromosome number by one. The transition from XY to neo-XY systems occurs due to a fusion between a sex chromosome and an autosome in the XY system to form a neo-XY system. In contrast, Y degradation in a neo-XY system can result in a karyotype that is cytologically indistinguishable from the ancestral condition, here represented as a return to the XY state.

We used the R package chromPlus to build models of chromosome number evolution (Blackmon et al., 2019). The Markov model we used allows haploid autosome number to increase or decrease by one, representing fusion and fission, respectively (Figure 1). Models were fit using the MCMC function from the R package diversitree with a broad exponential prior of 2 for applied to all parameters (FitzJohn, 2012). We note that though we use the terms fusion and fission several underlying processes at the molecular level can lead to net changes in chromosome number. However, given that our dataset is restricted to chromosome counts, modeling the net gain (fission) and loss (fusion) of chromosomes is the most direct and common approach for estimating the tempo of chromosome number evolution (Glick & Mayrose, 2014; Zenil-Ferguson et al., 2017). Our use of the terms “fission” and “fusion” throughout this paper refer to these model-inferred processes of chromosome number increase and decrease. For the remainder of the paper we use chromosome number to refer to the haploid autosome count. For the rate of chromosome number evolution, we examined the basic model which only considers fusions and fissions without sex chromosome system transitions. This model was run on four distinct datasets, first with all taxa and then individually on each of the three families (Scarabaeidae, Lucanidae, and Passalidae). To account for uncertainty in phylogeny, we fit our model using each of 100 phylogenetic trees and allowed the MCMC to run for 100 generations. In cases where a tip in the phylogeny had multiple differing records for chromosome number we randomly chose one of the values for each MCMC run. Most of the parameter estimates converged in just a handful of generations, but we conservatively discarded the initial 50% as burn-in. This process yielded a total of 5,000 estimates that represent the posterior distribution of the parameters in the model while accounting for both uncertainty in tip states and phylogenetic history. We compared the differences in chromosome number evolution rates between families using the mean of the parameters and 95% highest posterior density.

We define a neo-XY system as one from a fusion between an ancestral sex chromosome (X or Y) and an autosome in an ancestral XY system. This event, referred to as a sex chromosome-autosome (SA) fusion, reduces the haploid autosome number by one and increases the size of the sex chromosomes (a neo-X or neo-Y). In many Coleoptera, these rearrangements quickly resolve into a stable, simple neo-XY configuration through the loss of the punctiform, ancestral Y chromosomes (Bracewell et al., 2017). Our probabilistic model specifically estimates the rate of transitions from a simple XY system to a simple neo-XY system. Our model does not explicitly differentiate between X-autosome and Y-autosome fusions, and it does not account for other evolutionary pathways such as sex chromosome turnover or the formation of multiple sex chromosome systems (e.g., *Ellispoptera marutha* X_1_X_2_X_3_X_4_Y) (Galián et al., 2007). These systems were not reported in our dataset. Therefore, our analysis is focused on testing a key prediction regarding the rate of simple SA-fusions, a process directly testable with the available data. One potential limitation of this method is that a series of fissions and fusions in a short time could lead to an underestimate of the total number of fusions joining autosomes. We do not believe this is likely as we have a relatively densely sampled phylogeny and relatively low overall rates of fusion and fission relative to the branch lengths in our phylogeny.

### Fusions of sex chromosomes with autosomes

To understand if Scarabaeoidae exhibits more sex chromosomes and autosome fusions (SA-fusions) than expected by chance, we used the stochastic mapping function in the R package evobiR. Using these stochastic maps we calculated the total number of fusions and the number of SA-fusions to determine the empirical proportion of SA-fusions (Jonika et al., 2023). To calculate the expected proportion of SA-fusion we used the equation described by Anderson et al. (2020). This equation calculates the proportion of expected SA-fusions based on the number of autosomes and sex chromosome system. We calculated the weighted sum of expected SA-fusions, which consider the duration of each karyotype state across the inferred stochastic maps. This expected proportion of SA-fusions calculated from stochastic maps serves as a null distribution. We then compared the highest posterior density (HPD) credible interval of the empirical observation distribution and the null distribution. Non-overlapping 95% HPD intervals indicate that SA-fusion rates differ substantially from autosomal fusion rates, suggesting significant excess or paucity of SA-fusions.

## Results

### Data collection and phylogenetic inference

**Figure 2:**
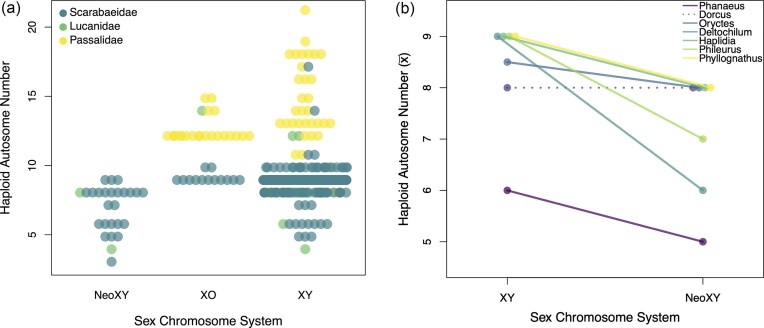
Patterns of chromosomal evolution in the superfamily Scarabaeoidea. (a) This figure visualizes all 478 available karyotype records from the database, showing the distribution of haploid autosome numbers and sex chromosome system (SCS). Each family is represented by a different color. The data points indicate the sex chromosome system (SCS) and haploid chromosome numbers for each taxon. The darker area indicates regions where data points are more densely clustered. The plot highlights the broad variation in both traits and the unequal distribution of SCSs among families. (b) There are 7 genera that possess both XY and neo-XY SCSs in available karyotype records from the database. The consistent decrease in autosome number in neo-XY lineages (solid lines) provides strong evidence the neo-XY systems in this group repeatedly arise from SA-fusions.

**Figure 3:**
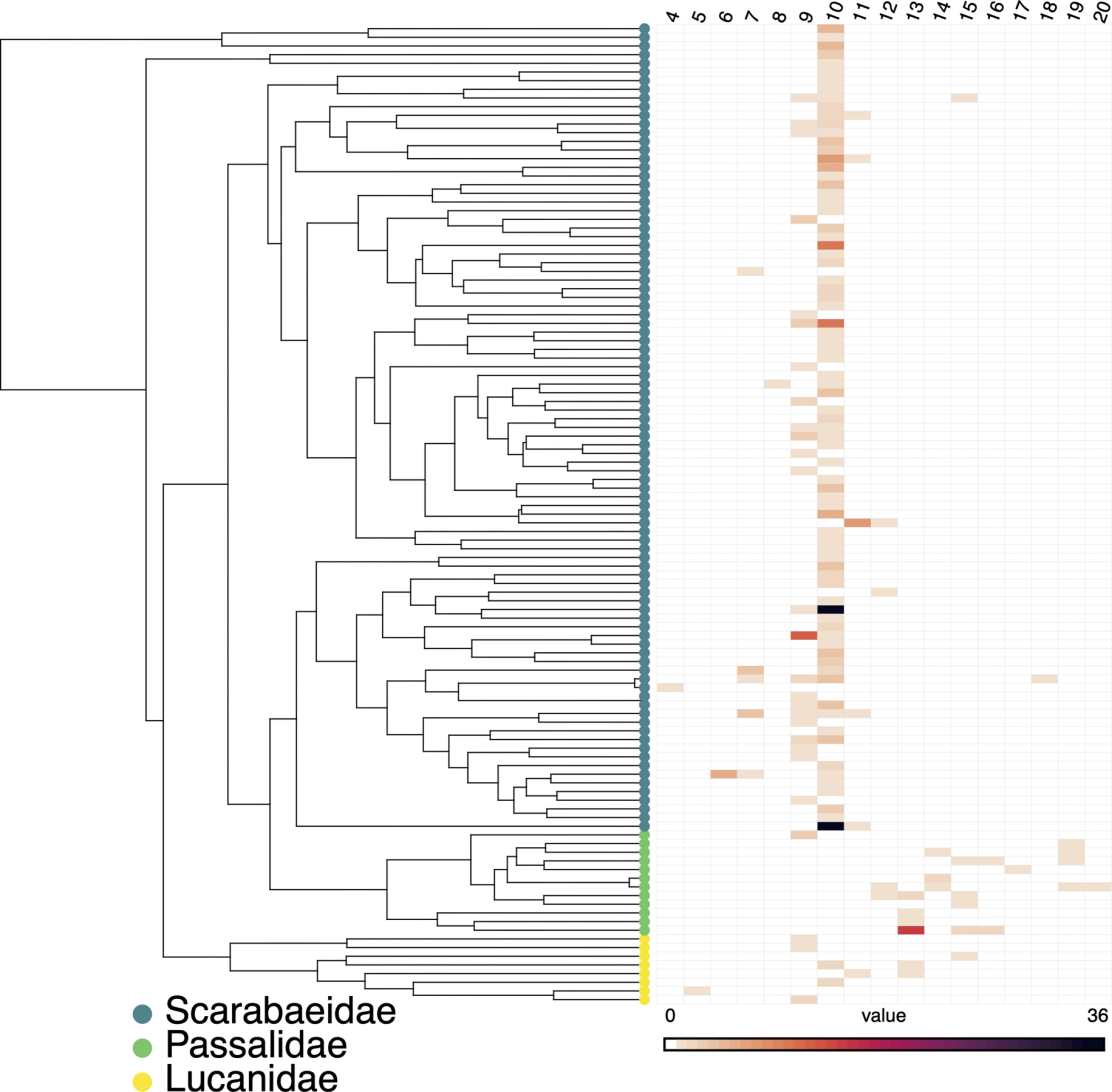
Haploid autosome number and species count across genera in Scarabaeidae, Passalidae, and Lucanidae. The distribution of haploid chromosome numbers is visualized across a phylogeny of 113 genera, which was collapsed to the genus-level from a larger species-level tree. In the accompanying matrix, haploid chromosome numbers are organized by column. The shading intensity of each cell is proportional to the number of species within a given genus (row) possessing that specific chromosome number.

We collected 478 karyotype records for Scarabaeoidae. The most common sex chromosome system was XY which was reported in 415 species. This was followed by XO and neo-XY, which were reported in 36 and 26 species, respectively (Figure 2A). Haploid autosome numbers ranged from a low of 4 in the Scarabaeidae *Eurysternus caribaeus* to a high of 21 in the Passalidae *Undulifer acapulcae* (Figure 2A) (Cabral-de-Mello et al., 2007; Serrano et al., 2009). We find a consistent pattern where species with neo-XY sex chromosome systems have fewer autosomes than species with an XY or XO sex-determination system. This pattern is consistent with frequent neo-XY origins from the fusions of an autosome and a sex chromosome (Figure 2B). We focused our comparative analyses on the families Scarabaeidae, Passalidae, and Lucanidae. Among these families, we were able to collect genetic data and build a phylogeny for 229 species. After removing the 18 taxa with phylogenetic placements that conflicted with their genus-level classification, 211 tips remain. These 211 species included 37 for which multiple and conflicting karyotype records existed (Figure 3).

### Rate of chromosome number evolution in scarabaeoidea

**Figure 4:**
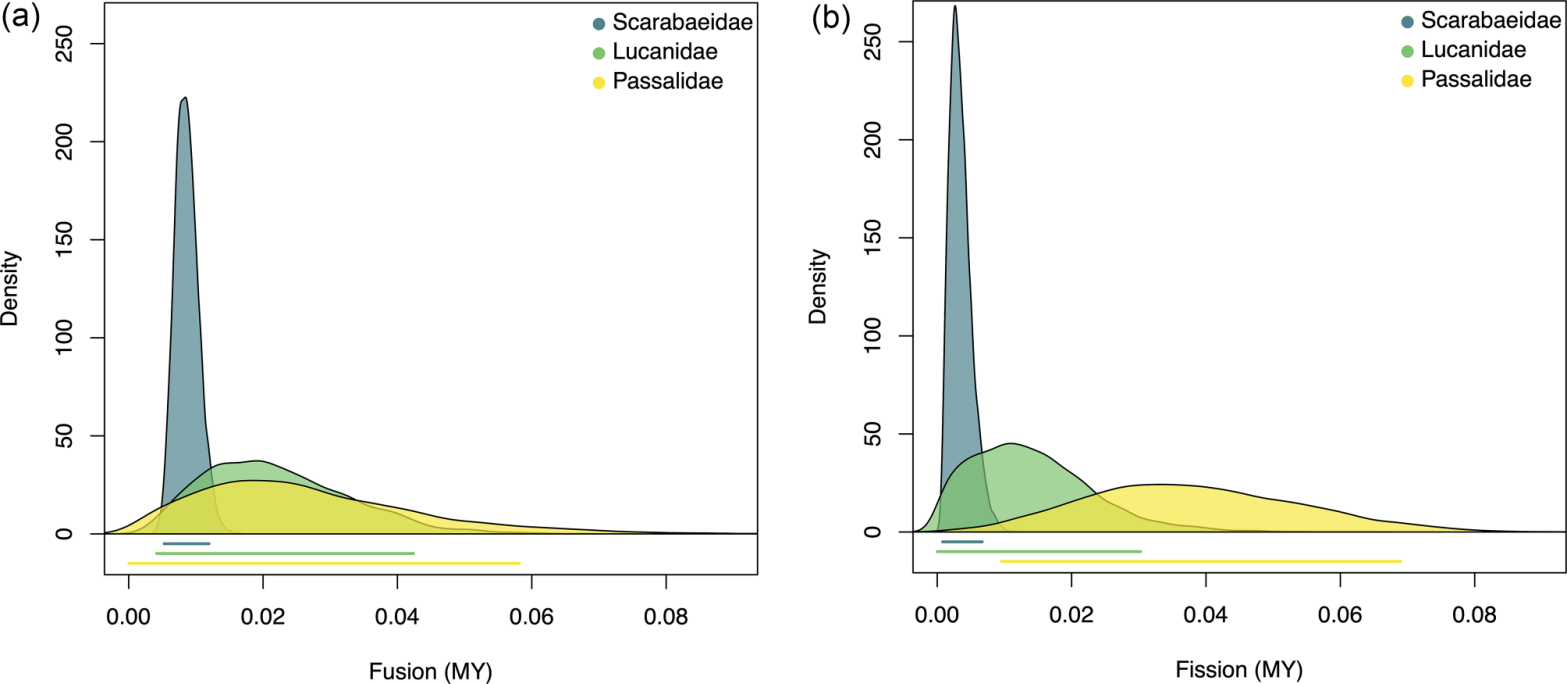
Posterior distribution of rates of fusions and fission in three Scarabaeoidae families. Each color represents a family. Panel A indicates rates of fusion and panel B indicates rates of fission. Passalidae has the highest rate of fusion and fission, and Scarabaeidae has the lowest rate of fusion and fission. The color bars below the distributions are the 95% HPD credible interval for each family. Non-overlapping 95% HPDs indicates a significant difference between the two families.

The MCMC run estimated the average autosomal fission rate of 0.006 and fusion rate of 0.007 per million years in the superfamily Scarabaeoidea. The 95% HPD credible intervals are overlapping, suggesting that fusion and fission rates are similar (Figure S2). Subsequent runs in each family show a similar ordering of rate magnitudes among families for both fusion and fission (e.g., Passalidae have the highest rate of both fusion and fission; Figure 4). The inferred mean rate of fusion is 0.009, 0.022, and 0.027 per million years in Scarabaeidae, Lucanidae, and Passalidae, respectively (Figure 4a). Because the 95% HPD of the estimated rates of fusions are overlapping, we infer that there is no significant difference among the three families. The mean rate of fission is 0.003, 0.014, and 0.038 per million years in Scarabaeidae, Lucanidae, and Passalidae, respectively (Figure 4b). The non-overlapping 95% HPDs indicate that Passalidae has a significantly higher fission rate than Scarabaeidae.

To test the robustness of our findings to the inclusion of genes-level data, we re-ran our analysis on a dataset of 174 species, for which sequence and karyotype data were derived from the same species. The overall pattern of evolutionary rates remained consistent with the main analysis. Passalidae continued to show the highest inferred mean rates of fusion and fission, while Scarabaeidae showed the lowest (Figure S3.) However, a key difference emerged for fission rates: with the reduced dataset, the 95% HPD intervals for fission rates now overlapped among all three families (Figure S3), indicating the previously observed significant difference between Passalidae and Scarabaeidae was no longer statistically significant. The 95% HPD intervals for fusion rates remained overlapping, as in the primary analysis. We interpret this as evidence for the importance of maximizing overlap between phylogenetic trees and comparative data and recommend the use of higher level tips in future studies.

### Fusions of autosomes with sex chromosomes

**Figure 5:**
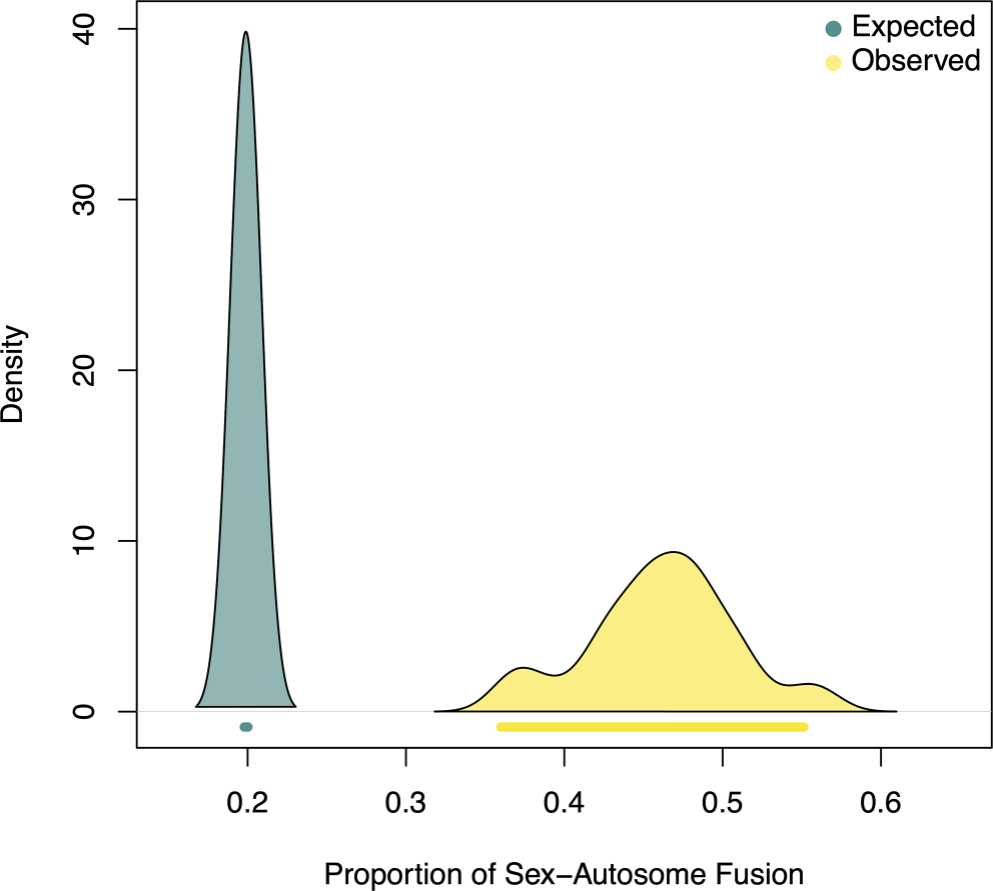
Posterior distribution of proportion of SA-fusion. Shade indicates the distribution of expected versus observed while bars underneath each distribution indicate the 95% HPD interval.

To test for an excess of SA fusions, we first examined the pattern on a representative tree from the posterior distribution. On this tree, the mean observed proportion of fusions that were SA-fusions was 0.46 (95% HPD: 0.36–0.55), which is significantly higher than the calculated null expectation of 0.19 (95% HPD: 0.19–0.20), as their 95% HPD intervals do not overlap (Figure 5). To confirm this result was not an artifact of tree choice, we assessed the outcome across all 100 posterior trees. We found a significant excess of SA-fusions (defined by non-overlapping 95% HPD intervals) in 86 of the 100 trees, demonstrating that the rate of fusions involving sex chromosome is significant higher than the background rate of autosomal fusions (as indicated by non-overlapping 95% HPD intervals). This finding is highly consistent and robust to phylogenetic uncertainty. Furthermore, our sensitivity analysis, which excluded genus-level data, confirmed this conclusion. To illustrate, on a representative tree from the posterior distribution of the reduced dataset, the observed proportion of SA-fusions was 0.30 (95% HPD: 0.24–0.37), which was significantly higher than the null expectation (mean: 0.20, 95% HPD: 0.196–0.199), which represents the rate of fusions that would be expected if chromosomal rearrangements occurred randomly across the genome, regardless of chromosome type (Figure S4). To ensure this was not an artifact of tree choice, we examined the results across all 100 posterior trees for the reduced dataset and found that a significant excess of SA-fusions was present in 80 of the 100 trees. This confirms that the conclusion of excess SA-fusions is a robust feature of karyotype evolution in Scarabaeoidea and not an artifact of including genus-level data.

## Discussion

The available karyotype data shows that the superfamily Scarabaeoidae exhibits striking variation in SCSs and chromosome numbers. 92% (24 of 26) neo-XY species are in the family Scarabaeidae, and there are no neo-XY reported in Passalidae. Interestingly, the XO system is prevalent in Passalidae, with 62% (23 of 37) of taxa possessing this system, representing 38% (23 of 60) of all taxa within the family. Notably, all members of the tribe Passalini within Passalidae display the XO system (Serrano et al., 2009). Passalidae have a higher mean haploid chromosome number than Scarabaeidae. Scarabaeidae typically maintains a conserved meioformula of 9 + Xy_p_. (i.e., 9 autosomes and an XX/XY sex chromosome system with a distance pairing punctiform y chromosome). The majority of taxa in Passalidae have haploid chromosome numbers above 14 (Blackmon & Demuth, 2015; Cabral-de-Mello et al., 2008; B. Dutrillaux et al., 2023). The variations in chromosome numbers and SCSs across superfamily Scarabaeoidae highlights the significant role that chromosomal rearrangements, such as fission and fusion, have played in the evolutionary history of the superfamily. These processes play an important role in recent evolution and speciation within the superfamily, particularly in Passalidae, where chromosomal changes consistent with multiple fissions have been observed (B. Dutrillaux et al., 2023).

Our results show that Passalidae have the highest rates of fission and fusion in Scarabaeoidae. This pattern is consistent with hypotheses linking life history traits to the tempo of karyotype evolution. Previous study suggests that beetles with a lower effective population size (N_e_) have higher rates of karyotype evolution likely due to genetic drift allowing for the fixation of underdominant or mildly deleterious structural changes (Blackmon et al., 2024; Jonika et al., 2024). Numerous Passalid beetles exhibit limited dispersal ability. Multiple species are characterized by reduced mobility, often associated with brachypterism (shortened wings), and are frequently confined to specific geographical areas, particularly in montane regions (Galindo-Cardona et al., 2007; Garrick et al., 2019; Jiménez-Ferbans et al., 2022). Therefore, the accelerated rates we observe in Passalidae are consistent with a scenario where genetic drift plays a more pronounced role in driving chromosomal change.

Our study has several limitations. First, matching karyotype and sequence data at the genus-level is a potential source of error. Our sensitivity analysis, which excluded these 37 taxa, demonstrated that our primary conclusions regarding the accelerated rates in Passalidae and the significant excess of SA-fusions are robust. However, the loss of statistical significance for the higher fission rate in Passalidae in the reduced dataset suggests this specific result should be interpreted with some caution, though we believe that it is likely due to reduced statistical power of the smaller dataset. Therefore, the relative conclusion that Passalidae has an accelerated tempo of karyotype change compared to the other families remains well-supported. Finally, as our study relies on karyotype data aggregated from the database and literature, it is potentially subject to sampling and publication biases. With regard to sampling, Scarabaeidae are most poorly sampled with approximately 0.5% of species that have been karyotyped. In contrast Passalidae has the most complete sampling with 1.5% of named species having been studied. Finally Lucanidae is intermediate between these. Future systematic surveys will be needed to generate a more complete and unbiased dataset to fully resolve these patterns.

Recombination can be suppressed by a variety of forces, including inversions, neutral divergence, or pre-existing recombination cold-spots (Jay et al., 2024; S. H. Smith et al., 2023), one of which is sexual antagonism (B. Charlesworth et al., 1987; B. Charlesworth & Wall, 1999). Recombination suppression is at the core of the canonical model of sex chromosome evolution (B. Charlesworth, 1991; Ironside, 2010; Lenormand & Roze, 2022). However, comparative studies to test whether patterns of chromosomal fusions across clades are consistent with the sexual antagonism hypothesis of Charlesworth and Charlesworth (D. Charlesworth & Charlesworth, 1980)have been limited by a lack of statistical frameworks. Recently, an approach was developed that allows comparative tests of one of the core predictions of the sexual antagonism hypothesis for sex chromosome evolution (Anderson et al., 2020). Our finding that SA-fusions are more common than expected by chance make Scarabaeoidae only the second animal clade where this pattern has been demonstrated using this probabilistic framework (Anderson et al., 2020). While our study utilizes this specific statistical approach, the role of sexual antagonism in driving sex chromosome evolution has been documented in other systems using diverse methodologies (Mora et al., 2024; D. A. S. Smith et al., 2016; S. H. Smith et al., 2023; Yoshida & Kitano, 2012). While this result is consistent with the canonical model where SA-fusions are selectively favored to resolve sexual antagonism (D. Charlesworth & Charlesworth, 1980), evolution of SA-fusions is a complex process likely impacted by multiple forces, including genetic drift, meiotic drive, sex-ratio selection and selection to expand the pseudoautosomal region to ensure proper pairing and avoid the loss of a degenerating sex chromosome (Pennell et al., 2015). The pronounced sexual dimorphism in Scarabaeoidae, is consistent with resolved sexually antagonistic selection. One possible pathway to the resolution of this sexual antagonism is SA-fusion that links the involved genes to the sex determining region. Scarabaeidae and Lucanidae exhibit some of the most characteristic cases of sexual dimorphism traits in Coleoptera, such as exaggerated male horn or mandibles used in mating competition (Darwin, 1871; Emlen et al., 2005). These traits are shaped strongly by sexual selection. The prevalence of such dimorphisms suggests that sexual antagonism may have played a prominent role in the evolution of sex chromosomes in this group.

Our findings suggest that Passalidae have higher fission and fusion rates than other families in Scarabaeoidae, a pattern consistent with the effects of their unique life history. However, this connection remains a hypothesis as our study did not formally model the influence of dispersal or effective population size. Future research could directly test this hypothesis by incorporating proxies for these traits into a phylogenetic comparative framework. Furthermore, to gain a deeper understanding of the factors driving these accelerated karyotype evolution patterns in Passalidae, we need to integrate high-quality genomic data with molecular cytogenetic and cytogenomic investigations. Such a multi-disciplinary approach will facilitate efforts to understand if rates of structural evolution at the gross scale are correlated with rates of evolution at the nucleotide scale. These additional resources would allow for a more comprehensive investigation into the mechanisms underlying the observed chromosomal dynamics.

## Supplementary Material

voag025_Supplemental_File

## Data Availability

All data and scripts necessary to replicate this study and figures are available at https://doi.org/10.5281/zenodo.19457363.
