# Supplementary material for: Chromosomal rearrangements: tempo and mode of karyotype evolution in Scarabaeoidea
Source: J Evol Biol. 2026 Apr 9;39(6):699–708. doi: 10.1093/jeb/voag025 (PMC13222848; doi:10.1093/jeb/voag025)
Supplement: voag025_Supplemental_File [file voag025_supplemental_file.docx]

## Supplementary material captions

##
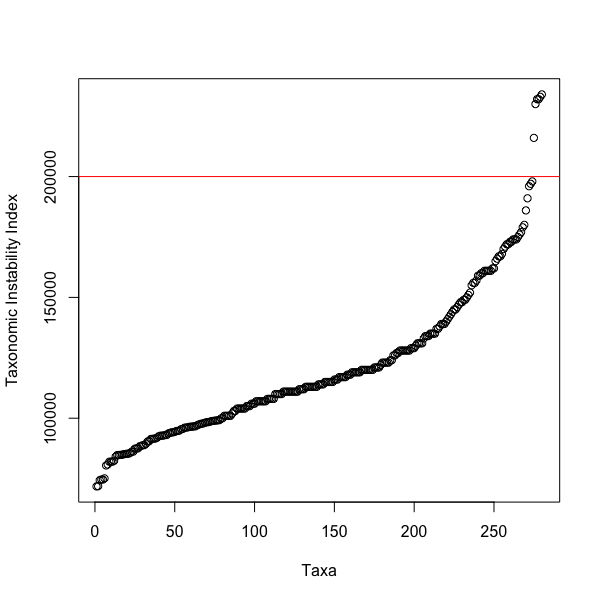

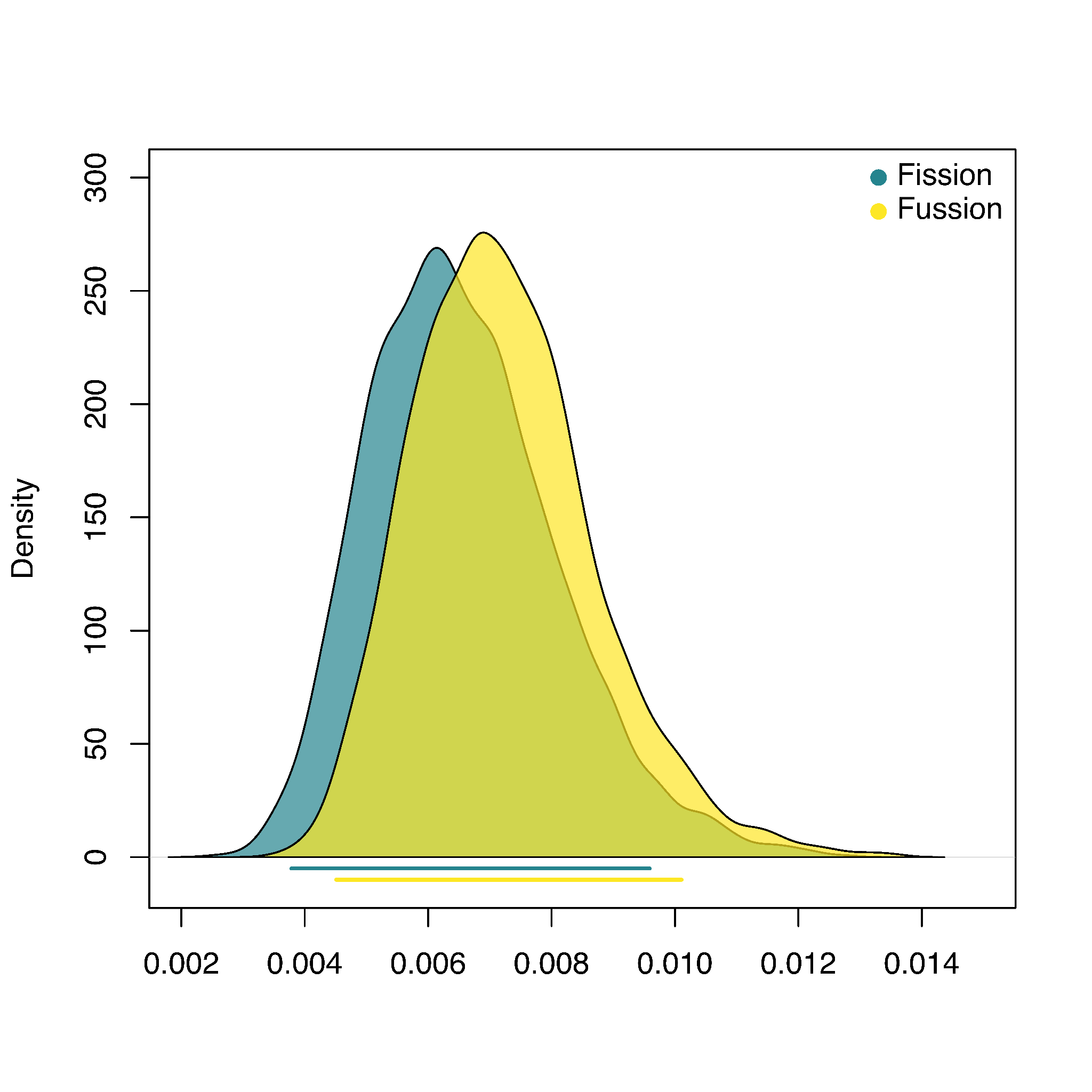


**Figure S1. Taxonomic instability indices.**

The red line indicates the cutoff of 20,000; 97% of taxa fall below this value, while TII increases quickly above it.

**Figure S2. Rate of fusion and fission in Scarabaeoidae.**

The overlapping HPD credible interval indicates there is no significant difference between the rate of fusion and fission in Scarabaeoidae, even though the rate of fusion has a higher average


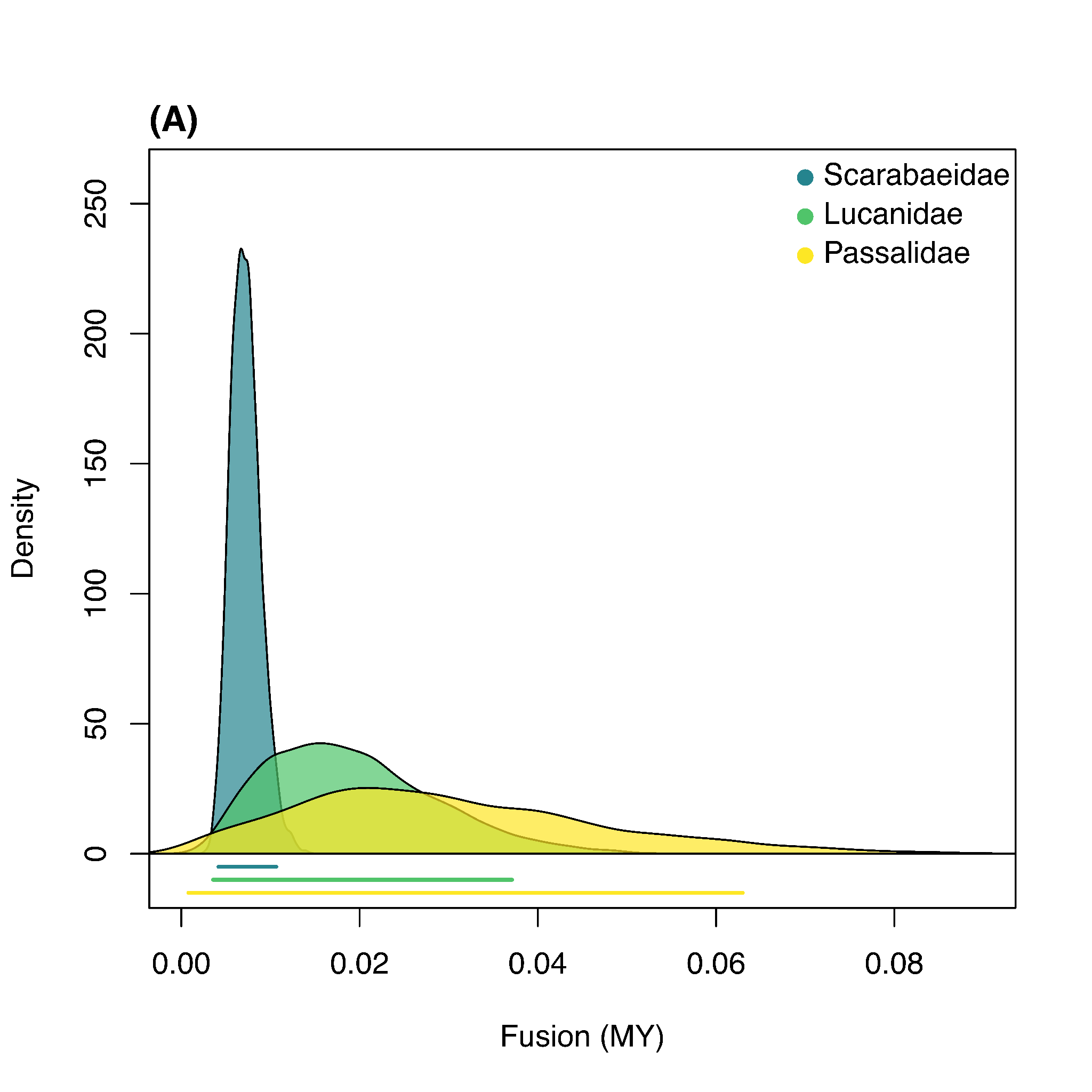

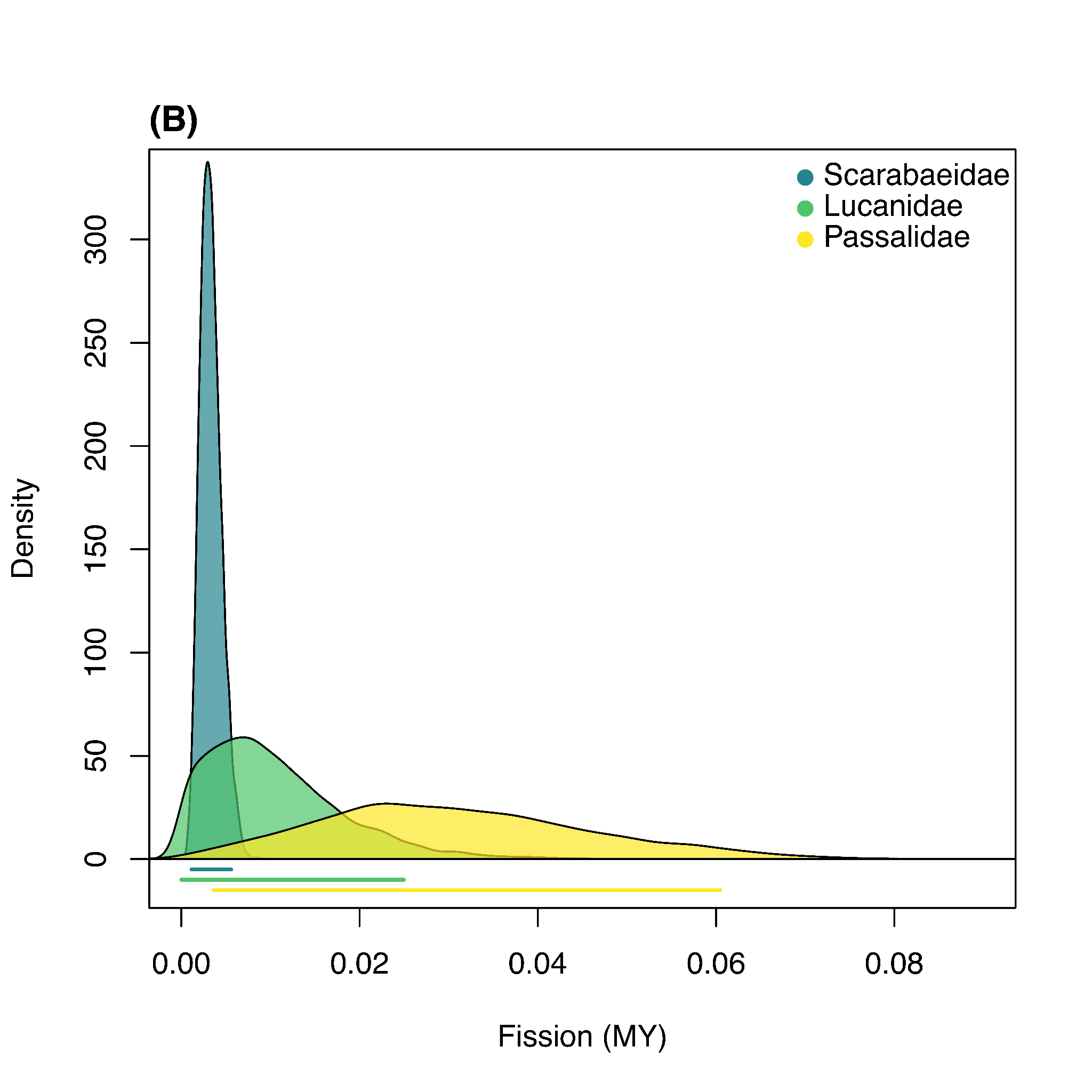


**Figure S3. Posterior distribution of rates of fusions and fission in three Scarabaeoidae families with only species-level match.**

These are the posterior distributions for (A) fusion and (B) fission rates estimated from the reduced dataset of 174 species, which excludes taxa with genus-level data matches. While the overall trend mirrors the primary analysis (Figure 5), with Passalidae exhibiting the highest mean rates, the key finding is that the 95% HPD intervals for fission rates now overlap among all families. This indicates that the significant difference in fission rates observed in the full dataset is no longer statistically significant with this more conservative sampling.

**
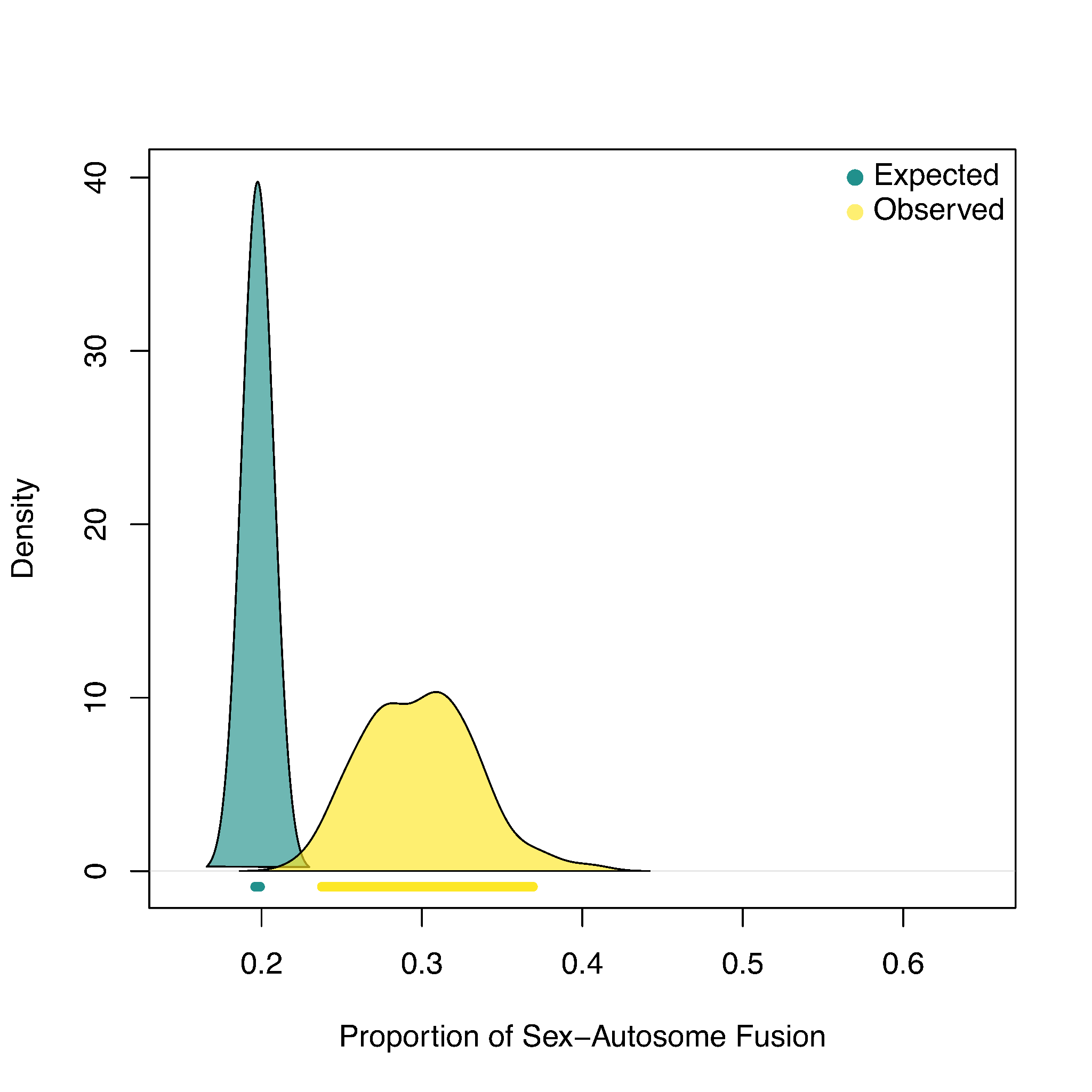
**

**Figure S4. Posterior distribution of proportion of SA-fusion.** Shade indicates the distribution of expected versus observed while bars underneath each distribution indicate the HPD interval. These are the posterior distributions from the reduced dataset of 174 species, which excludes taxa with genus-level data matches. While the overall trend mirrors the primary analysis (Figure 5)
